# 3D myocardial wall stress assessed by cardiac magnetic resonance and non invasive aortic blood pressure in patients with severe aortic valve stenosis

**DOI:** 10.1186/1532-429X-17-S1-P17

**Published:** 2015-02-03

**Authors:** Florence Pontnau, Gilles Soulat, Ludivine Perdrix, Valentina Zhygalina, Archid Azarine, Nadjia Kachenoura, Elie Mousseaux

**Affiliations:** 1 Cardiology, European Hospital Georges Pompidou, Paris, France; 2 Radiology, European Hospital Georges Pompidou, Paris, France; 3 Université Pierre et Marie Curie, Paris, France

## Background

To develop and evaluate a non-invasive 3D model of Myocardial Wall Stress (MWS) in subjects with severe aortic valve stenosis (AVS).

## Methods

We studied 20 subjects (71±10 years) with severe AVS including 19 with a preserved ejection fraction (EF) and 14 elderly controls (59±6 years). All subjects underwent within 4 hours, transthoracic echocardiogram (TTE) and cardiac magnetic resonance (CMR) as well as applanation tonometry of the carotid artery immediately after CMR examination. CMR included an ECG gated cine SSFP acquisition of left ventricular (LV) short axis slices, positioned regularly and without inter-slice gap between base and apex. Carotid tonometry, by adjusting by the mean brachial pressure obtained during CMR acquisition is a reliable measurement of central aortic blood pressure. 3D MWS can provide a LV afterload estimate which is well known to be strongly related to EF, except in case of depressed contractility. Evaluation of 3D MWS relied on the combination of: 1) a geometrical factor (GF), estimated according to myocardial thickness and LV cavity radius, while accounting for the 3D curvature of the LV, and 2) LV peak systolic pressure provided by tonometric measurement and Doppler maximal transvalvular pressure gradient.

## Results

For all patients, TTE revealed the presence of severe AVS according to ESC criteria (aortic valve area indexed to BSA= 0.43±0.09 cm2/m2 and mean gradient 54±14mmHg). When compared to 3D MWS evaluation of controls, GF indicated a significant decrease in patients with AVS (patients AVS 0.28±0.16; controls 0.37±0.13; p<0.05) whereas 3D MWS remained equivalent between the two groups. These data reflect LV adaptation to pressure overload, leading to an overall normalization of MWS in severe AVSwith preserved EF. Furthermore, our 3D model of MWS is strongly related to EF (r=0.84; p<0.05), reflecting the robustness of this non-invasive method based on CMR and applanation tonometry.

## Conclusions

This 3D non-invasive evaluation of MWS based on CMR and carotid tonometry was able to characterize LV adaptation to pressure overload in severe AVS. Since, MWS provides a LV afterload estimate, which is strongly related to EF, except in case of depressed contractility, future studies should be performed to reveal the particular usefulness of such non-invasive 3D MWS for prediction of recovery after aortic valve replacement in subjects with low EF.

## Funding

This study was funded by Assistance Publique dex Hôpitaux de Paris.

